# Essential Oils: Sources of Antimicrobials and Food Preservatives

**DOI:** 10.3389/fmicb.2016.02161

**Published:** 2017-01-16

**Authors:** Abhay K. Pandey, Pradeep Kumar, Pooja Singh, Nijendra N. Tripathi, Vivek K. Bajpai

**Affiliations:** ^1^Bacteriology and Natural Pesticide Laboratory, Department of Botany, Deen Dayal Upadhyay Gorakhpur UniversityGorakhpur, India; ^2^Department of Forestry, North Eastern Regional Institute of Science and TechnologyNirjuli, India; ^3^Department of Applied Microbiology and Biotechnology, School of Biotechnology, Yeungnam UniversityGyeongsan, South Korea

**Keywords:** essential oils, antibacterial, antifungal, food preservative properties, bioactivity

## Abstract

Aromatic and medicinal plants produce essential oils in the form of secondary metabolites. These essential oils can be used in diverse applications in food, perfume, and cosmetic industries. The use of essential oils as antimicrobials and food preservative agents is of concern because of several reported side effects of synthetic oils. Essential oils have the potential to be used as a food preservative for cereals, grains, pulses, fruits, and vegetables. In this review, we briefly describe the results in relevant literature and summarize the uses of essential oils with special emphasis on their antibacterial, bactericidal, antifungal, fungicidal, and food preservative properties. Essential oils have pronounced antimicrobial and food preservative properties because they consist of a variety of active constituents (e.g., terpenes, terpenoids, carotenoids, coumarins, curcumins) that have great significance in the food industry. Thus, the various properties of essential oils offer the possibility of using natural, safe, eco-friendly, cost-effective, renewable, and easily biodegradable antimicrobials for food commodity preservation in the near future.

## Introduction

**Figure 1:**
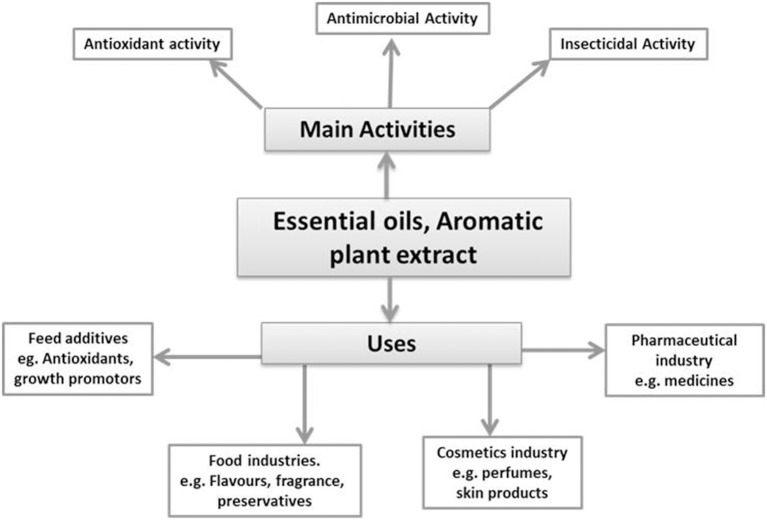
**Different activities and uses of essential oils**.

Since ancient times, commercial antimicrobial agents have been applied as a way to manage food deterioration or contamination. Nowadays, user concerns toward synthetic preservatives have resulted in increasing attention on various natural antimicrobials such as essential oils. Aromatic and medicinal plant essential oils and their components demonstrate antibacterial, antifungal, and food preservative activities against a wide range of microbial pathogens (Basim et al., 2000; Iacobellis et al., 2004; Tripathi and Kumar, 2007; Pandey et al., 2014b; Sonker et al., 2015; Gormez et al., 2016; Figure 1). These essential oils are hydrophobic liquids of aromatic compounds that are volatile and oily in nature and present in various plant parts such as twig, flower, leaf, bark, seed, and root. Many plant essential oils are useful as a flavor or aroma enhancer in cosmetics, food additives, soaps, plastics resins, and perfumes. Moreover, curiosity about essential oil applications that can act as antimicrobial agents is growing because of the broad range of activities, natural origins, and generally recognized as safe (GRAS) status of essential oils. Currently, essential oils are frequently studied for their antimicrobial (Cowan, 1999; Burt, 2004; Nedorostova et al., 2009), antifungal (Singh and Tripathi, 1999), antiulcer (Dordevic et al., 2007), antihelminthic (Inouye et al., 2001), antioxidant (Mimica-Dukic et al., 2003), anti-inflammatory (Singh et al., 1996), repellent, insecticidal, antifeedant (Isman et al., 1990; Pandey et al., 2014a), cytotoxic (Sylvestre et al., 2007), antiviral (Maurya et al., 2005), ovicidal (Pandey et al., 2011b), anesthetic (Ghelardini et al., 2001), molluscicidal (Fico et al., 2004), immunomodulatory (Mediratta et al., 2002), antinociceptive (Abdollahi et al., 2003), and larvicidal (Jantan et al., 2003) properties as well as for their use as food preservatives (Ukeh and Mordue, 2009; Pandey et al., 2014c).

Essential oils of aromatic and medicinal plants are reported to be effective against agents affecting stored products such as insects, human pathogenic fungi, and bacteria. Essential oils of *Chenopodium ambrosioides, Clausena pentaphylla, Mentha arvensis*, and *Ocimum sanctum* are contact-sensitive and act as fumigant toxicants against *Callosobruchus chinensis* and *C. maculatus* (Pandey et al., 2011a) associated with pigeon pea seeds. Similarly, the essential oil of *Tanacetum nubigenum* exhibit significant repellent and fumigant toxicity against *Tribolium castaneum*, which affects wheat during storage (Haider et al., 2015). *Eucalyptus globulus* essential oil has antibacterial activity against *Escherichia coli* and *Staphylococcus aureus*, thus, it is effective against both Gram-positive and Gram-negative bacteria (Bachir and Benali, 2012). In addition, other bacterial pathogens such as *Haemophilus influenzae, S. aureus, S. pneumonia*, and *S. pyogenes* were inhibited by *Eucalyptus odorata* essential oil under *in vitro* conditions (Posadzki et al., 2012). This review highlights the use of essential oils and their antifungal, fungicidal and food preservative properties in controlling fungi associated with food commodities. Additional emphasis has been given on the efficacy of essential oils against plant pathogenic bacteria as antibacterial and bactericidal.

## Essential oils and functions of their active constituents

**Figure 2:**
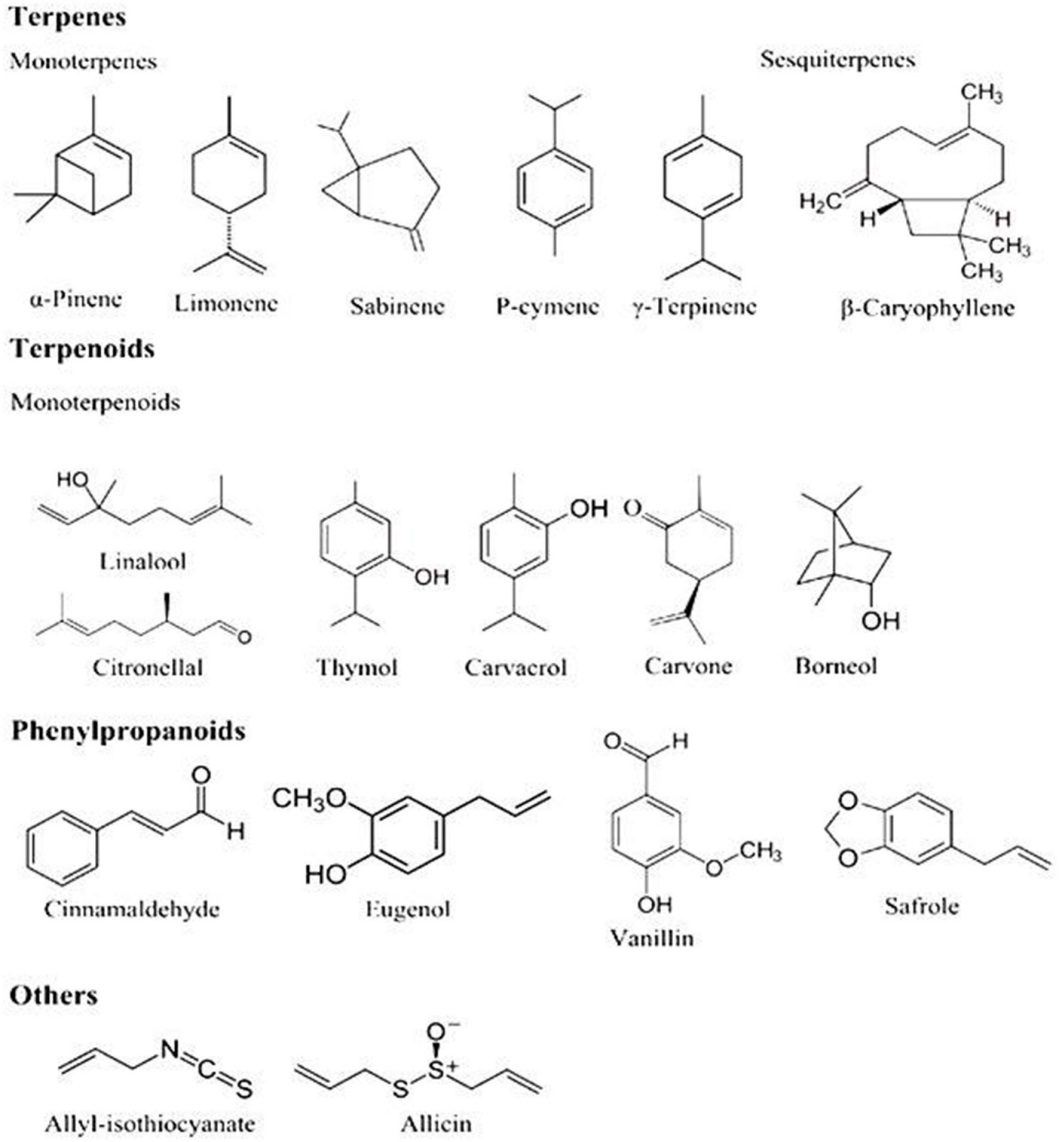
**Actives compounds of essential oils**. Figure as originally published in Hyldgaard et al. (2012).

The majority of aromatic plants retain a volatile odoriferous mixture of compounds which can be extracted as an essential oil. Generally, aromatic and medicinal plants produce a wide range of secondary metabolites *viz*., terpenoids, alcoholic compounds (e.g., geraniol, menthol, linalool), acidic compounds (e.g., benzoic, cinnamic, myristic acids), aldehydes (e.g., citral, benzaldehyde, cinnamaldehyde, carvone camphor), ketonic bodies (e.g., thymol, eugenol), and phenols (e.g., ascaridole, anethole). Among those, terpenes (e.g., pinene, myrcene, limonene, terpinene, *p*-cymene), terpenoids (e.g., oxygen-containing hydrocarbons), and aromatic phenols (e.g., carvacrol, thymol, safrole, eugenol) are found to have major roles in the composition of various essential oils (Figure 2) (Koul et al., 2008). Derivatives of terpenoids and aromatic polyterpenoids are synthesized by the mevalonic acid and shikimic acid pathways, respectively (Bedi et al., 2008). Terpenoids are among an immense pool of secondary compounds produced by aromatic and medicinal plants, and they have an important role in providing resistance to pathogens. Monoterpenoids are antimicrobial in nature, result in disruptive multiplication and development of microorganisms, and interfere in physiological and biochemical processes of microorganisms (Burt, 2004). Some botanical constituents such as azadirachtin, carvone, menthol, ascaridol, methyl eugenol, toosendanin, and volkensin have reported potential to act against several bacterial and fungal pathogens as well as against insect pests (Isman, 2006; Pandey et al., 2012, 2016). Moreover, many of them have powerful bactericidal, fungicidal, and insecticidal activities and can be responsible for improved taste or toxic properties.

Fungi such as *Aspergillus flavus, Neurospora sitophila*, and *Penicillium digitatum* are completely inhibited by *Cymbopogon citratus* essential oil (Shukla, 2009; Sonker et al., 2015). Essential oils from *Nigella sativa, Cymbopogon citratus*, and *Pulicaria undulata* inhibit the growth of *Bacillus subtilis, Staphylococcus aureus, Pseudomonas aeruginosa* and *Escherichia coli* (El-Kamali et al., 1998). Essential oils from *Acorus, Artemisia, Chenopodium, Clausena, Curcuma, Cinnamon, Cymbopogon, Eupatorium, Foeniculum, Hyptis, Lippia, Ocimum, Putranjiva, Syzygium*, and *Vitex* are known for their pronounced antimicrobial properties (Pandey et al., 2012, 2013b, 2014c; Sonker et al., 2015). The antibacterial properties of essential oils and their several active natural compounds against foodborne bacteria and their applications in food (Burt, 2004) could provide alternatives to conventional bactericides and fungicides (Perricone et al., 2015).

## Potency of essential oils against phytopathogenic bacteria

In cereals, pulses, fruits, and vegetables, bacterial species can cause major loss of plant quality and quantity during cultivation, transit, and storage by 20–40% of the total harvest per year. The bacterial species responsible for many diseases and loss of crops include *Clavibacter michiganensis, Pseudomonas syringae* pv. *tomato, P. solanacearum, P. cichorii, P. syringae* pv. *syringae, P. putida, Erwinia carotovora, E. amylovora, E. carotovora* subsp. *atroceptica, E. chrysanthemi, E. herbicola, Xanthomonas citri, X. campestris, X. axanopodis* pv. *malvacearum, X. axanopodis* pv. *vesicatoria, X. axanopodis* pv. *campestris, X. campestris* pv. *raphani, X. axanopodis* pv. *vitians*, and *X. campestris* pv. *zinnia*. Such bacteria cause substantial losses in many crops of national and international significance (Agrios, 2005). There are many essential oils that have been evaluated for their potential for antibacterial activity against these phytopathogenic bacteria under *in vitro* and *in vivo* conditions (Dorman and Deans, 2000; Iscan et al., 2003; Kotan et al., 2013). The methods used to assess the actions of essential oils against phytopathogenic bacteria include disc diffusion, agar dilution, agar well, and broth dilution (Perricone et al., 2015). Antimicrobial studies of essential oil constituents and their mode of actions more have been extensively undertaken on bacteria; however, there is limited information available about their actions on yeasts and molds.

Generally, Gram-negative bacteria are less susceptible to essential oils than Gram-positive bacteria. The outer membrane of Gram-negative bacteria contains hydrophilic lipopolysaccharides (LPS) that acts as a barrier to macromolecules and hydrophobic compounds, thus providing increased tolerance to hydrophobic antimicrobial compounds such as those found in essential oils (Nikaido, 1994, 2003; Trombetta et al., 2005). Therefore, it is difficult to predict the susceptibility of microorganisms to essential oils due to the breadth of genetic variations among species. Antibacterial activities of essential oils against a variety of phytopathogenic bacteria are summarized in Table 1.

**Table 1:** **Antibacterial investigations of essential oils against phytopathogenic bacteria**.

**Plant species (essential oil) tested**	**Plant pathogenic bacteria**	**Remarks**	**Investigators**
**1**	**2**	**3**	**4**
*Curcuma longa*	*Erwinia carotovora, Pseudomonas solanacearum, Xanthomons citri, Xa. malvacearum*	Oil exhibited efficacy to all bacteria at 1:10 dilution than 1:1000 dilution.	Banerjee and Nigam, 1978
*Carum copticum*	5 Bacteria	Oils exhibited variable degree of efficacy to test bacteria. Dethymolysed oil of *Carum copticum* was found to be most potent bacteriotoxicant.	Pandey et al., 1981
*Origanum vulgare, Satureja hortensis, Thymus vulgaris*	*Erwinia amylovora*	The test bacterium was found to be susceptible toward all the tested oils.	Scortichini and Rossi, 1989
*Ocimum basilicum*	*Pseudomonas putida*	Resistant to the bacteria.	Lachowicz et al., 1998
*Artemisia afra, Pteronia incana, Rosmarinus officinalis*	*Erwinia carotovora, Erw. chrysanthemi*	All three oils showed variable range of zone of inhibition.	Mangena and Muyima, 1999
*Thymbra spicata*	6 Bacteria	The oil showed variable MIC and MBC values against all test bacteria in contact and volatile phase.	Basim et al., 2000
*Thymus vulgaris, Pelarogonium graveolens*	*Erwinia carotovora*	*Thymus vulgaris* showed highest zone of inhibition, while *Pelarogonium graveolens* showed no inhibition against test bacteria.	Dorman and Deans, 2000
*Cinnamomum zeylanicum, Cymbopogon citratus*	*Erwinia amylovora, Erw. herbicola*	*Cinnamomum zeylanicum* showed highest toxicity against both bacteria, while *Cymbopogon citratus* was found to be least toxic.	Vanneste and Boyd, 2002
*Rosa damascena*	*Xanthomonas vesicatoria* XV(88.5, 56, 97.2)	Remarkably inhibited tested strains of *Xa. vesicatoria*.	Basim and Basim, 2003
*Satureja hortensis*	*Clavibacter michiganensis, Pseudomonas syringae* pv. *tomato, Xa. campestris* subsp. *campestris*	Oil was effective against all three tested organisms.	Gulluce et al., 2003
*Heracleum sphondylium* sub sp. *ternatum*	5 Bacteria	The MIC of oil was least against 3 bacteria (*Xa. compestris* pv. *phaseoli, Xa. compestris, Ps. syringae* pv. *syringae*) while higher against *Ps*. pv. *phasiolicola* and *Ps. syringae* pv. *tomato*).	Iscan et al., 2003
Thymol oil, Palmerosa oil, Lemongrass oil, Tea tree oil	*Ralstonia solanacearum*	In all the oil tested, 3 oils remarkably inhibited the growth of the test bacteria except Tea tree oil.	Pradhanang et al., 2003
*Rosa damascene, Thymbra spicata*	*Erwinia amylovora*	*R. damascene* oil was least effective with MBC value 1386.5 μg/m than *T. spicata* oil (500μg/m).	Basim and Basim, 2004
*Coriandrum sativum, Foeniculum vulgare*	27 Bacteria	A significant antibacterial activity was observed by agar diffusion method with *C. sativum* oil whereas a much reduced effect was observed for *F. vulgare* oil.	Cantore et al., 2004
*Coriandrum sativum, Foeniculum vulgare, Cuminum cyminum, Carum carvi*	29 Bacteria	A significant antibacterial activity was observed against Gram+ and Gram –ve bacteria. A much reduced effect was observed for the wild fennel.	Iacobellis et al., 2004
*Cuminum cyminum, Carum carvi*	31 Bacteria	The activity was particularly high against the genera *Clavibacter, Curtobacterium, Rhodococcus, Erwinia, Xanthomonas, Ralstonia* and *Agrobacterium* while lower activity was observed against *Pseudomonas* sp.	Iacobellis et al., 2005
Thymol, Palmarosa oil	*Ralstonia solanacearum*	Both Thymol and Palmarosa oil during soil treatment reduced bacterial wilt significantly.	Ji et al., 2005
*Artemisia absinthium, A. dracunculus, A. santonicum, A. spicigera*	16 Bacteria	Among all the 4 oils, *A. santonicum* and *A. spicigera* oils showed antibacterial activities over a very wide spectrum, ineffective against *Ps. syringae* pv. *populans*.	Kordali et al., 2005
*Ocimum gratissimum, Thymus vulgaris, Cymbopogon citratus, Zingiber officinale, Monodora myristica*	5 Bacteria	The essential oils from *Ocimum gratissimum* and *Thymus vulgaris* were highly effective against all bacteria tested, *Cymbopogon citratus* and *Zingiber officinale* were moderate effective while *Monodora myristica* was least effective.	Nguefack et al., 2005
*Artemisia turanica*	*Agrobacterium tumifacience*	The oil did not exhibit any antibacterial effect on test strains.	Behravan et al., 2006
*Thymbra spicata, Thymus kotschyanus, Origanum onites, Satureja hortensis*	*Clavibacter michiganensis* sub. sp. *michiganensis, Pseudomonas syringae* pv. *tomato, Xanthomonas campestris* pv. *malvacearum*	All the essential oils exhibited antibacterial activity against all pathogens except *Xa. campestris* pv. *malvacearum*. Gram+ve bacteria were more sensitive than Gram –ve bacteria.	Kizil and Uyar, 2006
*Ziziphora persica*	5 Bacteria	The oil showed variable range of zone of inhibition against *Ps. syringae* and *Erw. caratovora* while ineffective against other three bacteria.	Ozturk and Ercisli, 2006
*Origanum vulgare, Carum carvi*	6 Bacteria	*Origanum vulgare* exhibited highest effectiveness against all tested bacteria, *Xa. vesicatoria* 67 was the most sensitive to the essential oil extracted from *Carum carvi*.	Vasinauskiene et al., 2006
*Rosa brunonii*	*Xanthomonas campestris*	20% dilution of essential oil was found to be most effective.	Jangwan et al., 2007
24 Plants	*Xanthomonas axonopodis* pv. *vesicatoria*	Of 24 plant samples, 7 essential oils were highly active showing inhibition zone in range of 22–46.3mm and MIC of 25–200μl/ml in range.	Kotan et al., 2007
*Russowia sogdiana*	*Erwinia carotovora* var. *carotovora, Pseudomonas lachrymans, Xanthomonas vesicatoria, Agrobacterium tumifacience*	The oil exhibited a broad spectrum of antibacterial activity against all test bacteria with MIC values ranging from 0.2 mg/ml to 0.8 mg/ml.	Tan et al., 2007
*Satureja thymbra*	*Pseudomonas putida*	The treatment resulted in log reduction at the level below the detection limit formed for either 60 or 18 min.	Chorianopoulos et al., 2008
*Mentha arvensis, Citrus limonum, Tagetes bipinata, Lavandula latifolia*	*Clavibacter michiganensis* sub. sp. *sepedonicus* (cms), *C. michiganensis* sub. sp. *insidiosus* (cmi)	*Mentha arvensis* and *Citrus limonum* were effective against cms and *Tagetes bipinata* and *Lavandula latifolia* were effective against cmi.	Pouvova et al., 2008
13 Plants	*Agrobacterium tumefaciens, Erwinia carotovora* var. *carotovora*	All the oils exhibited variable degree of toxicity toward both bacteria.	Saad et al., 2008
*Teucrium montanum*	5 Bacteria	Oil exhibited toxicity. The diameters of zone of inhibition ranged from (10–18 mm) with the highest zone of inhibition were observed against *Azotobacter chlorococcum. Agrobacterium tumifaciens* and *Erwinia carotovora* showed higher level of resistant.	Vukovic et al., 2008
*Satureja hortensis*	*Erwinia amylovora*	The MIC and MBC of oil against *Erw. amylovora* were found to be 0.025 and 0.05 μl/ml respectively.	Mihajilov-Krstev et al., 2009
*Metasequoia glyptostroboides*	*Xanthomonas campestris* pv. *campestris* KC94-17, *Xa. campestris* pv. *vesicatoria* YK93-4, *Xa. oryzae* pv. *oryzae* KX019, *Xanthomonas* sp. SK12	The minimum inhibitory concentration (MIC) and minimum bactericidal concentration (MBC) values of oil and the extract were ranged from 125 to 250 μl/ml and 125–500 μg/ml and 250–1000 μl/ml and 250–2000 μg/ml respectively.	Bajpai et al., 2010a
*Cleistocalyx operculatus*	*Xanthomonas campestris* pv. *campestris* KC94-17, *Xa. campestris* pv. *vesicatoria* YK93-4, *Xa. oryzae* pv. *oryzae* KX019, *Xanthomonas* sp. SK12	The MIC and MBC values of the oil and extract against the tested *Xanthomonas* spp. ranged from 31.25 to 125 μl/ml and 62.5 to 250 μg/ml respectively.	Bajpai et al., 2010b
*Satureja hortensis, Thymus vulgaris*	*Erwinia amylovora*	20 μl dose of both oil exhibited 25mm zone of inhibition.	Karami-Osboo et al., 2010
*Origanum* sp., *Thymus* sp., *Mellisa* sp., *Mentha* sp. and *Nepeta* sp	*Erwinia amylovora, Pseudomonas syringae* pv. *syringae*	1 μl dose of all the oils exhibited maximum efficacy and *Origanum* sp. oil was found to be more potent.	Kokoskova et al., 2011
*Chenopodium ambrosioides*	*Erwinia herbicola, P. putida*	The MIC and MBC of the oil were 0.25 and 2.0 μl/ml for *Erw. herbicola*, and 0.12 and 1.0 μl/ml for *Ps. putida*, respectively.	Pandey et al., 2012
*Satureja hortensis*	*Clavibacter michiganensis* ssp. *michiganensis, Erwinia amylovora, Erw. carotovora* subsp. *atroceptica, Erw. chrysanthemi, Pseudomonas cichorii, Ps. syringae* pv. *syringae, Ps. syringae* pv. *tomato, Xanthomonas axanopodis* pv. *malvacearum, Xa. axanopodis* pv. v*esicatoria, Xa. axanopodis* pv. *campestris, Xa. campestris* pv. *raphani, Xa. axanopodis* pv. *vitians, Xa. campestris* pv. z*innia, Xa. axanopodis* pv. *pelargonii*	12.5 μl dose of essential oil gave maximum zone of inhibition against *Xanthomonas axanopodis* pv. *vitians* (50 mm) followed by *Xanthomonas axanopodis* pv. *campestris* (45 mm), *Xanthomonas axanopodis* pv. *pelargonii, Xanthomonas axanopodis* pv. *malvacearum* (42 mm), *Erwinia carotovora* subsp. *atroceptica* (41mm) while zone of inhibition was <40 mm for other bacterial species.	Kotan et al., 2013
*Eupatorium adenophorum*	*Erw. herbicola Ps. putida*	The MIC and MBC values were ranges of 0.25–4.0 μl/ml for the both bacterial species.	Pandey et al., 2014b
*Nepeta hindostana*	*Erw. herbicola Ps. putida*	The MIC and MBC values were 2 and 8 μl/ml for *Erw. herbicola* and 4 and >16 for *Ps. putida*.	Pandey et al., 2015
*Origanum rotundifolium*	20 plant pathogenic bacteria	The essential oil exhibits significant antibacterial effect against the test bacteria.	Gormez et al., 2016

## Potency of essential oils against storage fungi

Fungi can act as major destroyers of food commodities, including cereals, pulses, fruits, and vegetables, through the production of mycotoxins and render food unhealthy for human consumption by adversely affecting their nutritional value (Paranagama et al., 2003; Pandey et al., 2016). During storage, spoilage of stored food commodities is a chronic problem in tropical hot and humid climates. According to the FAO, foodborne fungal pathogens and their toxic metabolites can produce qualitative and quantitative losses of up to 25% of total agricultural food commodities throughout the world (Agrios, 2005). Fungal infection in food commodities results in a reduction of food quality, color, and texture as well as a reduction in nutrients present and physiological properties of food commodities (Dhingra et al., 2001). During infection, fungi can also produce mycotoxins, which can lead to famines in developing countries (Wagacha and Muthomi, 2008). With regard to molds, food contamination by *Alternaria, Aspergillus, Penicillium, Fusarium*, and *Rhizopus* spp. is of great significance because of the related health hazards and foodborne infections (Pandey and Tripathi, 2011). Hence, during storage and transit, prevention of fungal growth by essential oils could be a cost-effective approach to combat food losses. In recent years, throughout the world, the antifungal potential of essential oils is being considered significantly important (Baruah et al., 1996; Arras and Usai, 2001; Lalitha and Raveesha, 2006; Bosquez-Molina et al., 2010). The antifungal activities of essential oils are related to the associated disintegration of fungal hyphae due to the mono- and sesquiterpene compounds present in the essential oils. Moreover, essential oils amplify membrane permeability; as such compounds can dissolve in cell membranes and cause membrane swelling, thereby reducing membrane function (Dorman and Deans, 2000). Additionally, the lipophilic property of essential oils is responsible for their antifungal activity as that property gives them the ability to penetrate cell walls and affect enzymes involved in cell-wall synthesis, thus altering the morphological characteristics of the fungi (Cox et al., 2000). The present account summarizes the investigations into essential oils tested for their antifungal activity against fungi affecting food storage (Table 2).

**Table 2:** **Antifungal investigations of essential oils against fungi infecting food commodities during postharvest**.

**Plant species (essential oil) tested**	**Postharvest fungi**	**Remarks**	**Investigators**
**1**	**2**	**3**	**4**
*Raphanus sativus*	*Alternaria brassicae, Fusarium avenaceum, Phoma* spp.	The oil was active at 1:250 to 1:1000000 dilutions.	Nehrash, 1961
*Juniperus communis*	*Aspergillus niger*	Exhibited toxicity.	Slavenas and Razinskaite, 1962
*Eugenia bracteata, E. heyneana*	*Cephalosporium sacchri, Curvularia lunata, Fusarium moniliforme*	Both the oils were toxic toward test fungi.	Rao and Joseph, 1971
*Cymbopogon citratus, Mentha arvensis*, Sweet basil	*Penicillium italicum*	*Mentha* oil was most toxic during both *in vitro* and *in vivo* testing.	Arora and Pandey, 1977
*Curcuma aromatica, C. caesia, Myristica fragrans*	15 Storage fungi	*Myristica fragrans* was most toxic.	Kher and Chaurasia, 1978
*Trachyspermum ammi, Oenanthera stalonifera, Anethum graveolens, Apium graveolens, Parthenium histerophorus* and *Psoralea corylifolia*	16 Fungal species	The essential oils exhibited significant antimycotic activity.	Sharma and Singh, 1979
*Cestrum diurnum*	39 Storage fungi	The oil inhibited mycelial growth of all test fungi at 0.7% concentration.	Renu et al., 1980
*Ageratum conyzoides*	*Colletotricum capsici, Penicillium italicum*	The oil was found to be toxic at its MIC of 0.5% and 0.2% against *C. capsici* and *P. italicum* respectively.	Chandra and Dixit, 1981
*Ageratum conyzoides, Cymbopogo*n *martini* var. *motia, Eupatorium capillifolium, Ocimum adscendens*,	*Helminthosporium oryzae*	The oil of *Ocimum adscendens* was found most effective at 200 μl/l conc.	Asthana et al., 1982
*Citrus medica, Ocimum canum*	*Aspergillus flavus, A. versicolor*	Oils completely inhibited the mycelial growth of test fungi at 2000 ppm.	Dubey et al., 1983
*Alpinia galanga*	24 Storage fungi	The oil showed broad antifungal spectrum at 0.4% and 0.6% conc.	Tripathi et al., 1983
Lemon grass, *Mentha* sp., Palmarosa, *Zingiber* sp.	*Aspergillus parasiticus*	*Mentha* oil was most potent control the growth of *A. parasiticus* and aflatoxin production.	Kala et al., 1984
*Citrus aurantifolia*	20 Storage fungi	Fungitoxic at 2000 ppm.	Upadhyay et al., 1985
*Ocimum adscendens*	30 Storage fungi	Fungicidal at 400 ppm.	Asthana et al., 1986
*Anisomeles ovata*	*Aspergillus flavus*	Effective at 2000 ppm.	Upadhyay et al., 1987
*Eucalyptus* sp.	*Aspergillus niger*	Checked mycelial growth at 1000 ppm.	Tiwari et al., 1988
Thyme, Cumin, Clove, Caraway, Rosemary, Sage	*Aspergillus parasiticus*	All the oils exhibited broad range of fungitoxicity.	Farag et al., 1989
*Amomum subulatum*	*A. flavus*	Fungitoxic at 3000 ppm.	Mishra and Dubey, 1990
*Daucus carota*	*A. flavus*	Exhibited antifungal activity at 2000 ppm.	Dwivedi et al., 1991
*Cinnamomum camphora*	*A. flavus*	Fungitoxic at 400 ppm.	Mishra et al., 1991
*Ocimum gratissimum*	*A. flavus, A. niger*	MIC of oil was 100 ppm against *A. flavus* and 900 ppm against *A. niger*.	Dixit and Shukla, 1992
*Hyptis suaveolens*	21 Storage fungi	Exhibited mycelial inhibition at 2000 ppm.	Singh et al., 1992
Mixture of *Apium graveolens* and *Cuminum cyminum*	29 Storage fungi	Mixture showed antifungal activity at the conc. of 1:1 ratio.	Mishra et al., 1993
14 Plant essential oils	47 Storage fungi	*Cymbopogon citratus* oil was found to be fungistatic in nature at 1500ppm concentration against all test fungi.	Mishra and Dubey, 1994
*Cinnamomum zeylanicum*	35 Storage fungi	Showed strongest activity at 400 ppm.	Tiwari et al., 1994
*Nardostachys jatmansi*	*A. flavus, A. niger, F. oxysporum*	Completely inhibited mycelial growth of all test fungi at 1.0 × 1000 μl/l.	Mishra et al., 1995
*Cymbopogon martini, Eucalyptus citriodora, Cinnamomum tamala, Mentha piperita*	*F. moniliforme*	Mycotoxic activity of oils increased with increase concentration of oil.	Baruah et al., 1996
*Monarda citriodora, Melaleuca alternifolia*	15 Post-harvest fungi	Exhibited absolute toxicity.	Bishop and Thornton, 1997
*Thymus vulgaris*	*Botrytis cinerea, Rhizopus stolonifer*	Absolutely inhibited the mycelial growth of test fungi.	Reddy et al., 1998
*Cedrus deodara, Tracchispermum ammi*	*A. niger, Curvularia ovoidea*	The MIC of oils was found to be 1000 and 500 ppm respectively.	Singh and Tripathi, 1999
*Ocimum gratissimum, Zingiber cassumunar* and *Cymbopogon citratus*	*A. flavus*	Oils exhibited antimycotic activity at its MIC ranging from 500 to 1300 ppm.	Dubey et al., 2000
*Thymus capitatus*	*Alternaria citri, Botrytis cinerea, Penicillium digitatum, P. italicum*	Oil was effective against all test fungi at 250 ppm conc.	Arras and Usai, 2001
*Caesulia axillaris*	*A. flavus, A. niger*	MIC of oil against test fungi was 1000 ppm.	Dubey et al., 2002
*Putranjiva roxburghii*	15 Storage fungi	The MIC and MFC of the oil were 400 and 600 ppm respectively.	Kumar and Tripathi, 2002
Cinnamon and Clove oil	*C. musae, F. proliferatum, Lasiodiplodia theobromae*	The oils were effective at 500 ppm concentration.	Ranasinghe et al., 2002
*Acorus calamus, Hedychium spicatum*	*Helminthosporium oryzae, F. moniliforme*	The oil inhibited growth of test fungi at 0.5 × 10^3^ml/l and 1.0 × 10^3^ml/l respectively.	Mishra et al., 2003
*Cymbopogon citratus*	*A. flavus*	Oil was fungistatic and fungicidal nature at 0.60 and 1.0mg/ml concentration respectively.	Paranagama et al., 2003
*Cymbopogon flexuosus*	25 Fungi	Oil showed absolute toxicity against all fungi at 0.3 μl/ml conc.	Shahi et al., 2003
*Curcuma longa*	10 Storage fungi	The oil exhibited 10% toxicity at 3000 ppm.	Singh et al., 2003
*Chrysanthemum viscidehirtum*	*Botrytis cinerea, Phytophthora citrophthora*	*Chrysanthemum viscidehirtum* exhibited strong activity at 150 ppm.	Chebli et al., 2004
*Mentha piperita*	*P. digitatum*	*M. piperita* caused 100% inhibition at 1000 μg/ml conc.	Dhaliwal et al., 2004
9 Plant essential oils	*P. expansum*	All oils were found to be effective.	Neri et al., 2005
*Thymus vulgaris* and *T. copticum*	*Alternaria citri, Penicillium italicum, P. digitatum*	Out of 5 oils, *Thymus vulgaris* and *T. copticum* were absolutely fungitoxic at 500 mg/l conc.	Azizi et al., 2006
15 Plant essential oils	10 Fungi	13 essential oils were found to be effective inhibited mycelial growth of all test fungi at 3.0% (v/v).	Lalitha and Raveesha, 2006
Epicarp of *Citrus sinensis*	10 Post-harvest fungi	Oil exhibited absolute toxicity toward test fungi.	Sharma and Tripathi, 2006
*Mentha arvensis*	9 Post-harvest fungi	Oil exhibited absolute mycelial inhibition against *Aspergillus flavus, A. fumigatus, Helminthosporium oryzae* and *Sclerotium rolfsii* at 0.10 mg/ml.	Kumar et al., 2007
*Satureja hortensis*	*A. flavus*	Oil exhibited toxicity.	Dikbas et al., 2008
*Lippia scaberrima*	*Botryosphaeria parva, C. gloeosporioides*	Oil was found to be effective, absolutely inhibited the mycelial growth of test fungi.	Regnier et al., 2008
*Mentha arvensis*	*A. flavus*	The MIC of *Mentha* oil against *A. flavus* was recorded at 400μl/l and it exhibited broad fungitoxic activity against other 14 storage fungi.	Kumar et al., 2009
*Satureja hortensis, Thymus vulgaris*	*Botrytis cinerea*	Oil was found to be toxic and significantly inhibited the growth of test fungi	Abdollahi et al., 2010
Thyme and Mexican lime	*C. gloeosporioides, Rhizopus stolonifer*	0.060% concentration of thyme oil stopped the mycelial growth of both test fungi	Bosquez-Molina et al., 2010
Cinnamon oil	*C. musae*	0.4% concentration of oil suppressed mycelial growth	Maqbool et al., 2010
*Mentha arvensis, Ocimum canum*	*A. flavus, A. ochraceus, A. niger, A. terreus*	Both the oils exhibited significant growth of all the test fungi at 500 ppm concentration.	Pandey and Tripathi, 2011
Eucalyptus, Clove, Cinnamon, Nutmeg, Neem, Nirgudi, Karanj, Sesame	*A. flavus, A. niger, A. terreus, A. oryzae, A. fumigatus, Fusarium moniliforme, F. solani* and *Penicillium* sp.	At 50 μl concentration of Eucalyptus, Clove, Cinnamon, Nutmeg oils were more potent against these fungi and among these cinnamon was most potent where zone of inhibition observed was in range of 22.5–67.5 mm.	Shirurkar and Wahegaonkar, 2012
*Clausena pentaphylla*	*A. flavus, A. ochraceus, A. niger, A. terreus*	Oil exhibited absolute mycelial inhibition at 0.36 μl/ml and MIC and MFC values were 0.07 μl/ml against all the test fungi	Pandey et al., 2013a
*Chenopodium ambrosioides*	*A. flavus, A. ochraceus, A. niger, A. terreus*	Absolute mycelia inhibition for all the test fungi was found at 0.36 μl/ml	Pandey et al., 2013b
*Cymbopogon citratus*	*A. flavus, A. niger, A. ochraceus*	A 0.33 μl/ml dose of the oil caused 100% mycelial inhibition and MIC was reported to be 0.29 μl/ml against all the test fungi	Sonker et al., 2014
*Artemisia nilagirica*	*A. flavus, A. niger, A. ochraceus*	Oil showed absolute mycelial inhibition of all the test fungi at 0.33 μl/ml, and MIC and MFC reported were 0.29 and 0.58 μl/ml, respectively for all *Aspergillus* species.	Sonker et al., 2015
*Lippia alba*	*A. flavus*	Absolute mycelial inhibition was observed at 0.28 μl/ml. Oil was fungicidal (MIC) at 0.14 μl/ml, and fungistatic at 0.28 μl/ml.	Pandey et al., 2016

## Potency of essential oils in food preservation

Research into the utility of essential oils in the preservation of food commodities in order to enhance shelf-life has been successfully carried out in recent years. Various investigators have used essential oils, either in pure or formulation forms, to enhance the shelf-life of food commodities in different storage containers such as those made of cardboard, tin, glass, polyethylene, or natural fabrics and have observed significant enhancement of shelf-life (Tripathi and Kumar, 2007; Pandey et al., 2014a). An earlier study reported that some essential oil constituents such as citral, citronella, citronellol, eugenol, farnesol, and nerol could protect chili seeds and fruits from fungal infection for up to 6 months (Tripathi et al., 1984). Essential oil from *Ageratum conyzoides* successfully controlled rotting of mandarins by blue mold and increased mandarin shelf-life by up to 30 days (Dixit et al., 1995). Anthony et al. (2003) investigated essential oils from *Cymbopogon nardus, C. flexuosus*, and *Ocimum basilicum* and observed that they could significantly control anthracnose in banana and increased banana shelf-life by up to 21 days. *Cymbopogon flexuosus* essential oil (20 μL/mL) is capable of protecting against rotting of *Malus pumilo* fruits for up to 3 weeks (Shahi et al., 2003). An fumigant application of essential oils from *Putranjiva roxburghii* was effective against *A. flavus* and *A. niger* infecting groundnuts during storage and enhanced the shelf-life of groundnut from fungal biodeterioration for up to 6 months (Tripathi and Kumar, 2007). The use of *Cymbopogon pendulous* essential oil as a fumigant increased groundnut shelf-life by 6–12 months (Shukla, 2009), thus proving to be more effective than *P. roxburghii* essential oil. These differences in efficacy of essential oils may be related to the use of oils from different plant species, as well as to their chemical composition, dose level, and storage container type.

Thyme (*Thymus capitata*) (0.1%) and maxican lime (*Citrus aurantifolia*) (0.5%) oil reduced disease incidence in papaya fruit (Bosquez-Molina et al., 2010), while cinnamon (0.3%) oil extended the storage life of banana by up to 28 days and reduced fungal disease incidence in banana (Maqbool et al., 2010). Seed dressing and fumigation of *Ocimum cannum* oil (1 μL/mL) enhanced the self-life of *Bhuchanania* (Singh et al., 2011). *Clausena pentaphylla* and *Chenopodium ambrosioides* oils, when used as fumigants in glass containers and natural fabric bags were able to protect pigeon pea seeds from *A. flavus, A. niger, A. ochraceus*, and *A. terreus* infection for up to 6 months (Pandey et al., 2013a,b). Powder-based formulations of *C. pentaphylla* and *C. ambrosioides* oils were also able to preserve pigeon pea seeds for up to 6 months (Pandey et al., 2014c). *Artemisia nilagirica* oil as a fumigant in cardboard improved the shelf-life of table grapes by up to 9 days (Sonker et al., 2015). Similarly, *Lippia alba* oil when used as an air dosage treatment in glass containers inhibited fungal proliferation and aflatoxin production in green gram (*Vigna radiata*) and enhanced its shelf-life by up to 6 months (Pandey et al., 2016).

## Conclusion and future prospects

Worldwide investigations carried out on essential oils have motivated researchers to focus their interest toward the study of botanical antimicrobials. It is apparent that the use of essential oils and their derivatives has been widely described, and essential oils have been used against a wide range of pathogens. Accordingly, this review provides a brief overview of essential oils, their active constituents, and their potential as sources of antibacterials, antifungals, and food preservatives. The relevant literature summary shows that essential oils exhibit a diverse range of antimicrobial properties, and indicates their natural sustainability when used as potential biocontrol agents against fungal and bacterial pathogens. Hence, we conclude from this review that essential oils are potential sources of biocontrol products that should be further explored due to their potential to protect food commodities. Also, an essential oil-based fumigant having antimicrobial activity should have a promising GRAS status in mammalian systems. The LD_50_ values of some botanicals like azadirachtin and carvone are found to be high in rat and are reportedly nontoxic for human consumers. Additionally, several essential oils and their constituents (e.g., carvone, carvacrol, cinnamaldehyde, thymol, linalool, citral, limonene, eugenol, limonene, and menthol) are reported by the United States Food and Drug Administration to have a GRAS status and are approved as flavor or food additives.

Essential oil applications are evolving as a means of integrating pathogens into food containers; for example, fumigants that can be useful in natural fabric and cardboard containers, and even containers made of wooden boards. Some oils can be used as light sprays and integrated as a fumigant into the commodity itself. Many essential oils and their active constituents are active against bacteria and fungi, and they can be produced from commonly available raw materials; perhaps in many cases right at the site of use so as to be rather low-cost treatments. Based on this review, it can be summarized that it is possible to develop techniques for food commodity protection without the use, or with reduced use, of commercial bactericides and fungicides. Although the available literature indicates that essential oils are host specific, biodegradable, have limited effect on non-target organisms, have low levels of mammalian toxicity. There, sustainable and commercial uses have some drawbacks, such as their cost effectiveness. Regardless, there are innumerable potential uses of essential oils and more research is needed to meet the needs of a food industry shifting toward the use of green technology.

## Author contributions

AP, PS, and NT conceived and designed the experiments. AP performed the experiments. AP and PK write the manuscript and PK and VB did the editing. All the authors read and approved the final manuscript.

### Conflict of interest statement

The authors declare that the research was conducted in the absence of any commercial or financial relationships that could be construed as a potential conflict of interest.
